# Photon-counting CT outperforms dental CBCT in detecting small accessory canals in root-filled teeth in a phantom study

**DOI:** 10.1038/s41598-025-24439-0

**Published:** 2025-10-27

**Authors:** Stephan Rau, Alexander Rau, Friederike Lang, Anna Fink, Jakob Weiß, Elias Kellner, Fabian Bamberg, Britta A. Jung, Rainer Schmelzeisen, Martin Pichotka, Markus Altenburger, Maximilian Frederik Russe, Wiebke Semper-Hogg

**Affiliations:** 1 Department of Diagnostic and Interventional Radiology, Medical Center–University of Freiburg, Faculty of Medicine, University of Freiburg, Hugstetter Str. 55, 79106 Freiburg, Germany; 2 Department of Neuroradiology, Medical Center–University of Freiburg, Faculty of Medicine, University of Freiburg, Freiburg, Germany; 3 Department of Orthodontics, Medical Center–University of Freiburg, Faculty of Medicine, University of Freiburg, Freiburg, Germany; 4 Department of Oral and Maxillofacial Surgery, Medical Center–University of Freiburg, Faculty of Medicine, University of Freiburg, Freiburg, Germany; 5 Division of Medical Physics, Department of Diagnostic and Interventional Radiology, Medical Center–University of Freiburg, Faculty of Medicine, University of Freiburg, Freiburg, Germany

**Keywords:** Tomography, X-ray computed, Photon-counting CT, Photon-counting detectors, CBCT, Diseases, Health care, Medical research

## Abstract

Accurate visualization of dental structures in root-filled teeth is critical for endodontic assessment but challenging due to artifacts from root canal (RC) fillings. This study compares photon-counting-detector CT (PCD-CT) and cone-beam CT (CBCT) for detecting small accessory canals in the presence of RC fillings. A phantom model with four bovine teeth containing RC fillings and five artificial accessory canals (1000–200 μm) per tooth was embedded in a bovine rib. The phantom was scanned with clinical PCD-CT (ultra-high-resolution protocol) and dental CBCT (endodontic protocol). Canal visibility was evaluated using signal-to-noise criteria, and canal diameters were compared with micro-CT measurements, serving as the reference. Blooming artifacts from RC fillings were quantified using the full width at half maximum (FWHM) of attenuation profiles. PCD-CT detected all canals down to 400 μm and 81.5% at 200 μm, while CBCT reliably detected canals ≥ 800 μm, with detection dropping to 47.2% at 400 μm and none at 200 μm. Measurement errors for canal diameters were lower with PCD-CT. Artifact widths varied between modalities, with no clear superiority. PCD-CT provided higher detectability and measurement fidelity for small accessory canals in root-filled teeth compared to a dental CBCT, with similar filling-related blooming, supporting the potential of PCD-CT in post-treatment endodontic imaging.

**Supplementary Information:**

The online version contains supplementary material available at 10.1038/s41598-025-24439-0.

## Introduction

 Dental root canal (RC) treatment is a common procedure to preserve teeth with infected or irreversibly inflamed pulp. Successful endodontic treatment relies on the thorough debridement, disinfection, and obturation of the entire RC system, encompassing not only the main canals but also lateral components such as isthmuses, fins, accessory canals, and apical ramifications^1,2^. Especially undetected RCs or lateral canals and apical ramifications are of particular significance in endodontic pathology and treatment prognosis of RC treated teeth. Untreated or missed accessory canals and isthmuses often harbor residual bacterial infections, representing a major cause of persistent endodontic disease, flare-ups, and subsequent treatment failure^3–5^. Studies have shown that inflammatory tissue within these structures can persist after treatment, potentially contributing to persistent or newly emerging periradicular inflammation^5–7^. Given that primary root canals typically measure approximately 330–370 μm in diameter, while accessory canals, obliterated or calcified canals, and microfractures are often significantly smaller^8,9^, clinicians require high-resolution imaging to accurately visualize these fine structures during both pre- and post-treatment endodontic assessments to ensure and validate successful outcomes.

Cone-beam computed tomography (CBCT) has been widely adopted in endodontics for its ability to provide three-dimensional visualization of dental structures^10,11^. However, CBCT’s effectiveness can be compromised by artifacts, particularly in root-filled teeth^12,13^. Even though some dental CBCT units nominally support voxel sizes as small as 75–80 μm, the true spatial resolution feasible in clinical routine is usually much lower. Indeed, the practical spatial resolution in CBCT ranges roughly from 100 μm to 500 µm^14,15^, which implies that small hypodense structures like untreated or accessory canals (often below 200–300 μm) may not be reliably distinguished on CBCT images^16^. In this respect, the presence of RC sealers and fillers is of particular importance, as they lead to beam hardening and blooming artifacts, which potentially obscure fine details and thereby hinder image assessment^17^.

Photon-counting-detector CT (PCD-CT) is an emerging technology with the potential to overcome some of these challenges. Unlike conventional energy-integrated-detector CTs, photon-counting detectors use a direct conversion semiconductor layer to convert X-ray photons to electrical signals. This innovation dramatically reduces electronic noise and allows for smaller detector pixels, thereby enabling higher intrinsic spatial resolution with an effective resolution of approximately 150–200 μm in clinical scanners^18,19^. In the dental domain, initial reports are similarly encouraging: PCD-CT has been shown to produce excellent visualization of dental structures^20–22^. Moreover, PCD-CT may mitigate common artifacts by reducing beam-hardening and metal artifacts^23^, which could be advantageous for imaging post-treatment teeth that often contain radiopaque filling materials or metal posts. To date, there is a lack of systematic comparative studies on the effects of root canal filling materials on the detectability and measurement accuracy of small accessory canals between PCD-CT and CBCT, leaving a clinically relevant gap in imaging performance after treatment.

Therefore, the aim of this phantom study was to further evaluate and compare the ability of a clinical PCD-CT with a dental CBCT in visualization of small artificial canals serving as exemplary small dental structures in root-filled teeth. The null hypothesis stated that PCD-CT and dental CBCT do not differ in accessory canal detectability, canal width measurement accuracy, or blooming artifacts.

## Materials and methods

### Phantom preparation

In an established experimental set-up^16^, a bovine rib and four bovine teeth were used to construct a jaw phantom model^16^, whereas in this setup the teeth were treated with RC fillings (ProTaper Next, Guttapercha, AH+, Dentsply Sirona, Bensheim, Germany). Bovine teeth were chosen due to their anatomical and morphological similarity to human incisors and their reduced variability compared to human specimens, which is widely accepted as substitutes in dental imaging research^24^. Their consistent size and structure help ensure reproducibility in phantom-based studies. Moreover, bovine ribs have been frequently utilized in endodontic imaging studies to simulate jawbone anatomy in CBCT accuracy assessments, particularly in RC length determination^25–27^. For this study, tissue from dead cows from the local slaughterhouse was utilized. The use of the tissues was approved by the local veterinary authority (City of Freiburg im Breisgau; Office for Public Order – Veterinary Department, AZ: 32.602.01, June 19th, 2024).

Phantom preparation was performed in four stages: First, following extraction, each tooth was first thoroughly cleaned and embedded in kneadable silicone to ensure stable positioning during the drilling process. To standardize drilling and prevent off-axis deviation, the labial root surfaces were flattened using a histological grinding device (Struers GmbH, Willich, Germany). Second, RCs were enlarged according to the standard procedure for root canal preparation in clinical routine (ProTaper Next, X-Smart plus, Dentsply Sirona, Bensheim, Germany). The main RCs were subsequently filled with RC filling material (Guttapercha, AH+, Dentsply Sirona, Bensheim, Germany). Third, five artificial accessory canals were drilled perpendicular to the axis of each root. Diameters of the accessory canals were 1000 μm, 800 μm, 600 μm, 400 μm, and 200 μm. Drilling was performed perpendicular to the flattened labial root surface to ensure consistent positioning and prevent drill deviation. All drill diameters were verified using a caliper with 1 μm resolution (Mitutoyo Europe GmbH, Neuss, Germany). Last, following preparation, the four filled teeth were finally embedded into a section of bovine rib using implant drills (Straumann GmbH, Freiburg, Germany) to simulate the anatomical configuration of a human mandible. The rib provided a rigid and anatomically analogous support structure. The phantom was stored in a humidified environment in a fridge (5–8 °C) to preserve the integrity of the specimens and prevent dehydration.

### Imaging systems

The phantom was scanned using three imaging modalities: a dedicated dental CBCT system, a clinical PCD-CT, and a high-resolution micro-CT system, which served as the reference standard. Scans were repeated three times in three varying gantry positions for both the CBCT and PCD-CT systems to assess reproducibility and reduce the influence of positional variability. For CBCT, the phantom was aligned in-plane with the scan direction; for PCD-CT, the phantom was oriented perpendicular to the scanner’s z-axis, mimicking typical head and neck scan positioning.

1. Dental CBCT: In a prior phantom study, we included two commercially available, state-of-the-art dental CBCT systems including the 3D Accuitomo 170, Morita, Osaka, Japan (80 μm voxel size) and the Planmeca Viso G7, Planmeca, Helsinki, Finland (150 μm voxel size). The systems showed similar performance in detection of small artificial canals using in small-FOV endodontic modes, while the 150 μm device exhibited higher image homogeneity. Noteworthy, the nominal voxel size does not equal practical spatial resolution in CBCT systems, as literature reports in vitro CBCT resolution to be at approximately 0.1–0.5 mm while systems allow for reconstructions with voxel sizes of down to 75 µm^14,15,28^. The spatial resolution is believed to be primarily determined by system mean transit function, reconstruction, detector characteristics, and motion rather than nominal voxel size alone. Accordingly, for this study we selected the Planmeca Viso G7 system (Planmeca, Helsinki, Finland) with scanning parameters in accordance to established single tooth imaging with voxel size of 150 μm. Additionally, default metal artifact reduction algorithm (ARA™, Planmeca, Helsinki, Finland) and adaptive image noise filter (AINO™, Planmeca, Helsinki, Finland) were used. Tube voltage was 100 kVp and tube current was 9.0 mA with 99.99 mAs. The scan time was 11.11 s.
2. Clinical PCD-CT: A first-generation clinical PCD-CT scanner (NAEOTOM Alpha, Siemens Healthineers, Forchheim, Germany) was used, applying a standard clinical protocol for ultra-high-resolution imaging of the temporal bone with the following parameters: tube voltage 120 kVp, IQ Level 110, tube current 28 mAs, pitch 0.85 and a scan time of 2.50 s.
3. Micro-CT: A micro-CT scanner (Bruker SkyScan 1276, Kontich, Belgium) served as the reference standard with an acceleration voltage 100 kVp, camera type XIMEA MH110XC-KK-TP with a pixel size of 17.468 μm. For data analysis, the pixel size was binned by a factor of (4) The scan was performed using a beam-filter combination of Al 0.5 and Cu 0.03 mm and an exposure time of 1,900 ms per step with rotation steps of 0.4°. The full scan comprises four 360° acquisitions, covering the full length of the jaw phantom.

### Image reconstruction and analysis

All acquired scans were reconstructed using dedicated software provided by the respective manufacturers. CBCT datasets were reconstructed with a filtered back-projection algorithm and a vendor-specific Feldkamp-Davis-Kress kernel with a field-of-view was 30 mm x 50 mm and a voxel size of 150 μm. The PCD-CT scan was reconstructed in axial series using a standard high-definition bone kernel (Hr80) with 0.2 mm slice thickness, 0.1 mm increment, 1024 × 1024 matrix size and a field-of-view of 100 mm x 100 mm with Quantum Iterative Reconstruction (QIR) strength 3. Micro-CT images were reconstructed with NRecon (Bruker, Kontich, Belgium) using beam hardening and ring artifact correction.

**Fig. 1:**
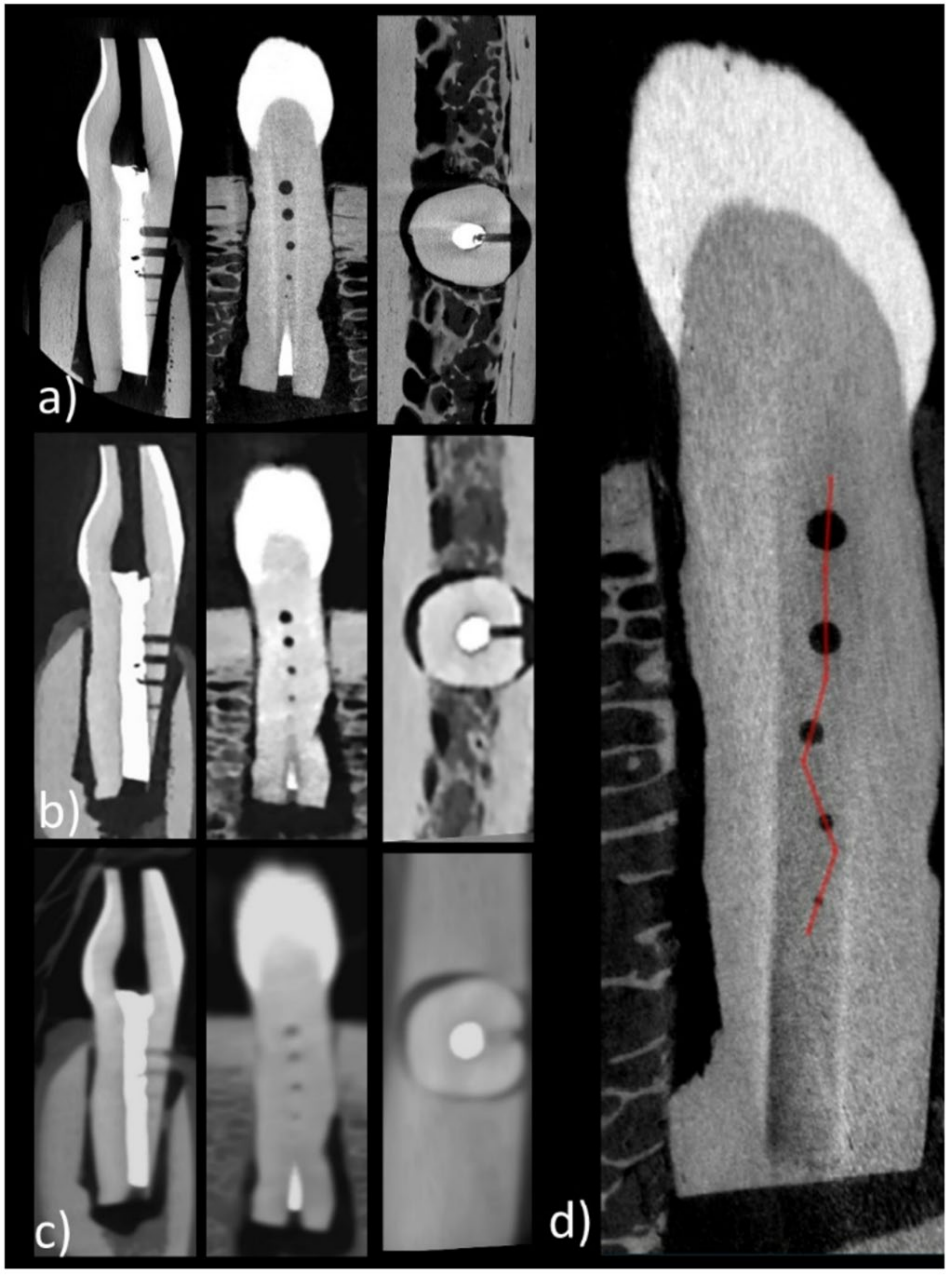
Representative multiplanar reconstructions with line profile aligned with the artificial accessory canals using tooth 1 as an example. The first row displays images acquired with micro-CT (**a**), the second row shows images from the photon-counting-detector CT (**b**), and the bottom row presents images obtained with cone-beam CT (**c**). The right image shows the placement of the line profile for this tooth obtained from the micro-CT (**d**).

1. Detection of Artificial Accessory Canals: Reconstructed DICOM datasets were imported into the publicly available post-processing platform NORA (NORA Medical Imaging Platform, Freiburg, Germany). Line profiles were placed by a radiologist with five years in head and neck imaging (SR) orthogonally through the center of each artificial accessory canal for each scan using multiplanar reconstructions. The visibility of each canal was assessed by comparing signal intensity in the canal to the surrounding noise. Canals were considered visible if their negative peak signal exceeded two times the standard deviation (2 × SD) of the background noise, measured from adjacent regions of interest (ROI). As the artificial canals are hypodense compared to dental tissue, the minimum intensity is used as a reference for this calculation, rather than the maximum. Therefore, canal width was measured using the full width at half density-minimum relative to the baseline tooth density and compared to the ground truth widths derived from micro-CT (Fig. 1, d).
2. Evaluation of Blooming Artifacts: Blooming artifacts caused by the RC fillings were evaluated using a two-point line profile through the filled main canal, centered at the mid-height between the first and second accessory canals, and in the exact direction of the canal with the largest diameter (1000 μm). Four equidistant ROIs were placed in surrounding dental material to determine local noise and allow for obtaining the ground level for the subsequent full width at half maximum (FWHM) analysis. The FWHM of the filling signal intensity was calculated based on the attenuation of the line profile and the determined ground noise level. Quantifying this width facilitates a comparative evaluation of blooming artifacts across different scanners. This evaluation was repeated for each tooth in both CBCT and PCD-CT datasets.

### Radiation dose

The radiation dose was measured using the dose-area product (DAP) for the CBCT scans and volume CT dose index (CTDI_vol_) as well as the dose-length product (DLP) for all PCD-CT-scans. To improve comparability, we converted the DAP of the CBCT scans into an equivalent CT-DLP using the region-specific conversion coefficient from Ketelhut et al. with k ≈ 22 cm for facial bones^29^.

$$\:K=\frac{DAP}{DLP}.$$

### Statistical analysis

Descriptive statistics were used to summarize the measurements, including canal width, detection rates, and blooming artifact dimensions. The accuracy of artificial canal width measurements was assessed by calculating absolute and relative error (%) against micro-CT reference values. Detection rates were calculated as the percentage of visible canals per diameter per modality. Blooming artifact widths based on FWHM of RC fillings were compared between PCD-CT and CBCT for each tooth individually using the Wilcoxon signed-rank test. All values for the artificial accessory canal detection, the width of artificial accessory canals and the width of fillings were reported as median values with interquartile range [IQR] of each tooth in all available scans. For canals detected in both modalities, medians of absolute error of canal width (paired by canal and modality) were taken and compared using the paired Wilcoxon signed-rank test. Median paired differences (PCD-CT minus CBCT) with 95% bootstrap confidence interval (1000 resamples) were reported. Test–retest precision was quantified from width measurements in nine repeats per canal (three repositions × three measurements) using the within-canal standard-deviation, within-canal coefficient of variation, and repeatability coefficient (repeatability coefficient = 1.96·√2·within-canal standard deviation). A p-value < 0.05 was considered statistically significant. All analyses were conducted using Python (version 3.10.12).

## Results

### Imaging acquisition and phantom visualization

**Fig. 2:**
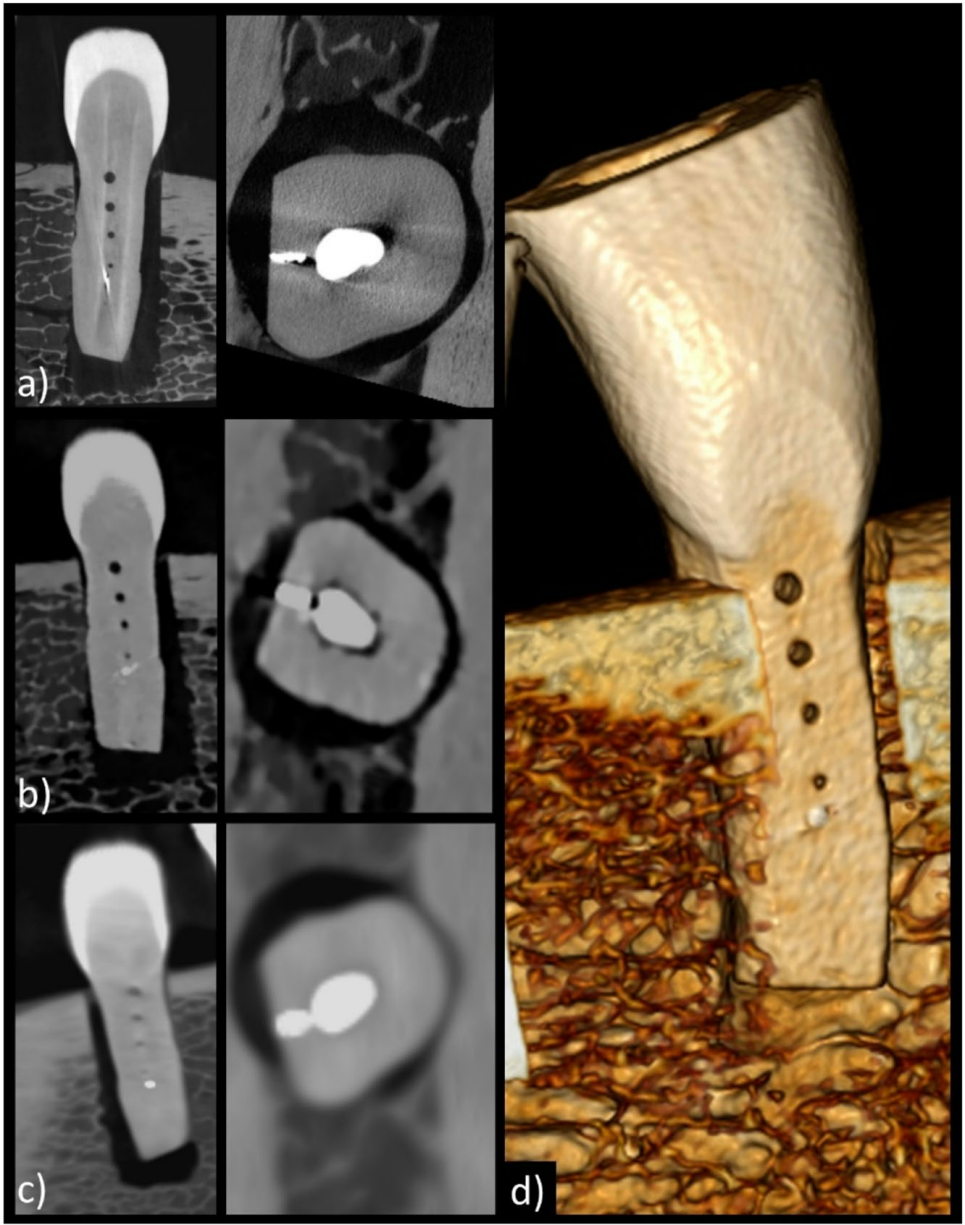
Multiplanar reconstructions in coronal and axial orientations of the smallest artificial accessory canal in tooth 2. The first row shows images acquired using micro-CT (**a**), the second row displays images from the photon-counting-detector CT (**b**), and the bottom row presents images from cone-beam CT (**c**). The volumetric reconstruction (**d**) was generated from the photon-counting-detector CT scan. Notably, the smallest artificial accessory canal contains a fractured drill tip. While increased beam hardening artifacts are visible in the PCD-CT images, the canal’s cylindrical shape is notably distorted in the CBCT images due to blooming.

Imaging acquisition was successfully completed in all modalities (CBCT, PCD-CT, and micro-CT) for all four teeth embedded in the jaw phantom. A representative example of image reconstructions from PCD-CT and CBCT is shown in Fig. 1, illustrating the visibility of artificial accessory canals of varying diameters as well as an exemplary line profile. In one specimen (Tooth 2), the drilling of the smallest accessory canal (200 μm) was unsuccessful due to breakage of the drill tip, which remained lodged within the tooth structure. This is visualized in Fig. 2, and the corresponding canal was excluded from the detectability analysis at this diameter for that tooth.

### Detection of artificial accessory canals

PCD-CT demonstrated superior performance in visualizing artificial accessory canals compared to dedicated dental CBCT (Table 1). PCD-CT achieved 100% detection rates for canals with diameters of 1000 μm, 800 μm, 600 μm, and 400 μm, and a high detection rate of 81.5% for 200 μm canals. In contrast, CBCT achieved 100.0% detection for canals with diameters ≥ 800 μm, which declined notably to 83.3% at 600 μm, 47.2% at 400 μm and 0.0% at 200 μm.

**Table 1:** Detection rate, relative and absolute error of artificial accessory Canals by imaging modality and canal diameter.

Canal diameter (µm)	Modality	Detection rate (%)	Relative error [IQR] (%)		Absolute error [IQR] (µm)	
1000	PCD-CT	100	2.9	[1.1, 4.9]	28	[10, 46]
	CBCT	100	16.1	[11.5, 20.1]	159	[112, 194]
800	PCD-CT	100	3.2	[2.2, 5.5]	25	[17, 44]
	CBCT	100	21.4	[13.9, 30.7]	169	[112, 246]
600	PCD-CT	100	4.6	[2.5, 7.9]	25	[14, 44]
	CBCT	83.3	14.7	[9.2, 23.6]	81	[52, 138]
400	PCD-CT	100	15.0	[8.5, 19.9]	60	[30, 79]
	CBCT	47.2	19.4	[6.5, 38.0]	80	[26, 134]
200	PCD-CT	81.5	95.1	[39.6, 163.1]	147	[60, 269]
	CBCT	0	n/a		n/a	

CBCT – cone beam CT; PCD-CT – photon-counting-detector CT.

### Measurement accuracy of canal widths

**Fig. 3:**
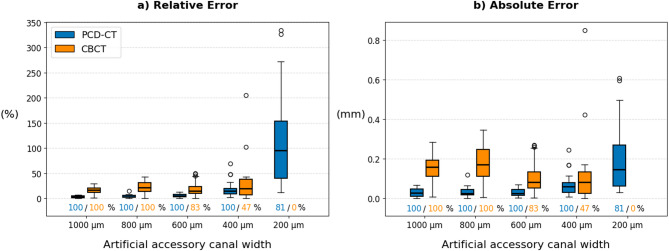
Comparison of measurement error in artificial accessory canals between PCD-CT and CBCT. Boxplots depict the relative error (**a**) and absolute error (**b**) for each artificial accessory canal width (200–1000 μm). Boxes indicate the interquartile range (IQR; 25th–75th percentile), with the median marked by a black line. Whiskers extend to the most extreme data points within 1.5 × IQR from the quartiles. Outliers beyond this range are plotted individually as points. Detection rates for PCD-CT and CBCT are annotated below. PCD-CT generally demonstrates lower error values and higher detection sensitivity across decreasing canal diameters.

Canal width measurements were compared to ground truth values obtained from micro-CT. PCD-CT exhibited lower measurement errors across all canal sizes (Fig. 3). Medians and IQR of relative errors are presented in Table 1.

For PCD-CT, relative errors remained below 4.6% for canals ≥ 600 μm and were 15.0% at 400 μm. At 200 μm, the error increased to 95.1%, reflecting expected challenges at the subvoxel level. CBCT exhibited higher variability and larger errors across all sizes, especially at 400 μm (19.4%) and 800 μm (21.4%). Absolute errors followed the same trend (Table 1). PCD-CT median absolute errors ranged from 25 μm to 60 μm for canals ≥ 400 μm and increased to 147 μm for canals with a diameter of 200 μm. CBCT errors were significantly higher, particularly for mid-range canal sizes (e.g., 169 μm vs. 25 μm in PCD-CT at 800 μm), and no values could be obtained for 200 μm canals due to lack of detection. In canals detected by both modalities (*n* = 15), absolute error of PCD-CT remained significantly lower with a median paired difference (PCD-CT − CBCT) of − 0.083 mm (95% CI − 0.190 to − 0.056; Wilcoxon *p* < 0.001). Repeatability was sub-voxel for both, PCD-CT (median within-canal standard-deviation 0.029 mm; repeatability coefficient 0.079 mm; within coefficient of variation 4.2%) and for CBCT (0.072 mm; 0.201 mm; 14.4%).

### Blooming artifact evaluation

**Fig. 4:**
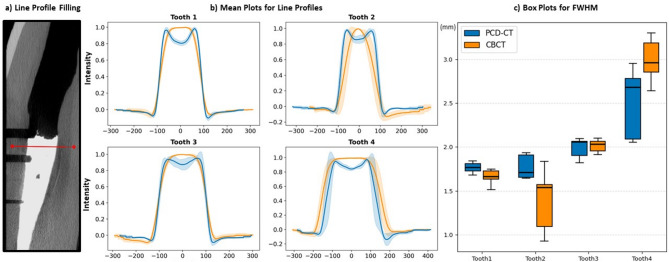
Line profiles and full width of half maximum (FWHM) of filling width measurements in photon-counting-detector CT (PCD-CT) and cone-beam CT(CBCT). First, line profiles were plotted parallel to the direction of the first artificial accessory canal as a reliable landmark at the mid-level between the first and second canal (**a**). Second, the mean attenuation values of the line profiles across the main root fillings were plotted for PCD-CT and CBCT (**b**). Shaded areas indicate ± 1 standard deviation around the mean. Each subplot corresponds to a tooth (Tooth 1–4). Third, boxplots were computed to show the FWHM from standardized measurements of the main root fillings in millimeters for each tooth (**c**).

Blooming artifacts associated with RC fillings were quantified by measuring the FWHM of the filling attenuation (Table 2; Fig. 4). The results demonstrate that the sizes of the filling are similar between PCD-CT and CBCT, suggesting that the blooming of the material did not lead to a one-sided overestimation of the filling width. In detail, in Tooth 1 and Tooth 2 PCD-CT exhibited significantly higher filling-width indicating more blooming-induced width-distortion compared to CBCT (*p* = 0.0039 and *p* = 0.0078, respectively), whereas in Tooth 4, CBCT demonstrated the opposite effect with a significantly higher filling-width compared to PCD-CT (*p* = 0.0273). For Tooth 3, no statistically significant difference between filling widths was observed between the scanners (*p* > 0.99).

**Table 2:** Width measurements of the artificial main root fillings compared between CBCT and PCD-CT.

	Tooth 1	Tooth 2	Tooth 3	Tooth 4
PCD-CT [mm]	1.76 [1.73, 1.81]	1.76 [1.66, 1.91]	2.00 [1.90, 2.07]	2.53 [2.09, 2.78]
CBCT [mm]	1.66 [1.63, 1.72]	1.39 [1.09, 1.57]	2.01 [1.95, 2.06]	3.01 [2.86, 3.19]

CBCT – cone beam CT; PCD-CT – photon-counting-detector CT.

### Radiation dose

For the PCD-CT, the median CTDI_vol_ was of 4.85 [4.84; 5.04] mGy and the DLP was 41.2 [41.2; 43.0] mGy*cm. The median DAP was 274 mGy*cm² for the CBCT scans. Using the facial-bones coefficient from Ketelhut et al., CBCT-DAP values of corresponded to an equivalent DLP of 12.45 mGy*cm.

## Discussion

The results of this phantom study indicate that PCD-CT significantly improves the visualization of small dental structures compared to dedicated dental CBCT, particularly in the context of root-filled teeth. PCD-CT consistently allowed reliable detection and precise measurement of artificial accessory canals simulating dental RCs down to 200 μm in diameter, clearly outperforming CBCT. In this setting, PCD-CT provided substantially more accurate measurements of canal diameters, with considerably lower deviations from ground truth compared to CBCT. Noteworthy is that the evaluation of detectability of accessory canals did not rely on a subjective reading but rather on an objective approach that relied on comparing the signal drop within the canal against the dental background. Furthermore, the artifact and noise profiles of PCD-CT with improved spatial resolution were comparable to those observed in high-end dental CBCT scans, as evidenced by the similar blooming artifacts surrounding the dental fillings.

Beyond the systematic quantitative evaluation in this study, analysis of the given image reconstructions (Figs. 1 and 2) on a merely subjective image level reveals an improved resolution of the surrounding bony structures with a more clearly defined microstructure in PCD-CT vs. CBCT.

As precise visualization of small anatomical structures in the range of 350 μm and smaller is of crucial importance in endodontics. The current clinical standard CBCT appears to be inadequate due to the limited spatial resolution of up to around 400–600 μm. In our previous phantom study, conducted without filling materials, the performance of the same dental CBCT in canal detectability was higher. In that study, CBCT could reliably detect canals with a diameter down to 300 μm and still allowed for the detection of canals with a diameter of 200 μm in 89%^16^. Besides the introduced inherent resolution constraints of CBCT, material-induced image artifacts such as blooming are a major issue in limiting image quality and image noise levels^14,15^. Noteworthy, the material composition is also of importance, as the type of RC filling materials influences the extent of blooming artifacts in CBCTs. AH + is one of the most widely used and well-studied root canal sealers worldwide and is based on epoxy resin. Among different root canal sealers examined, AH + generated the highest level of artifacts in dental imaging^12,13,17^. Nevertheless, the analysis of the blooming artifacts revealed no evident advantage of PCD-CT. In CBCT imaging, reconstruction choices and post-processing (e.g., metal-artifact-reduction algorithm, artifact suppression) materially influence depiction of fine structures: several recent reports show blooming reduction with specific software workflows, albeit sometimes with concomitant smoothing that may obscure small structures^30–33^. Our CBCT protocol used vendor-default metal-artifact-reduction algorithm and noise filter, which likely reduced blooming but also effective in-plane resolution, which is consistent with our results. Therefore, the impaired detection of the canals in the setting of the current study suggests that the lower canal detection and thus the reduced resolution is attributable to the presence of RC filling material.

In contrast, PCD-CT could demonstrate nearly consistently high image quality and canal detectability between both the current and the previous study with a reliable detection of the canals down to 400 μm and still very high detection rates of the smallest canal of 200 μm with currently 81.5% vs. 100% in the previous study without RC filling. Also, the relative error of the width measurement of the canals was only slightly reduced in the presence of fillings. Here too, the largest deviations were observed for the smallest hole, namely up to 95.1% compared to 85.6% in the previous study. In summary, PCD-CT’s advanced detector technology effectively mitigates these artifact-induced limitations, preserving diagnostic accuracy in challenging dental scenarios^18,21^. Moreover, PCD-CT offers enhanced artifact reduction from metal restorations, a frequent clinical obstacle encountered with CBCT^23,34^. This advantage is especially relevant in patient populations with complex dental restorations, enabling more accurate assessments of peri-implant bone and adjacent anatomical structures. Of particular interest in this context is the broken 200 μm drill tip shown in Fig. 2, which is stuck in the tooth structure. While more beam hardening artifacts can be seen in PCD-CT, the cylindrical shape is lost in CBCT due to blooming. The enhanced capability of artifact-reduced imaging of metal-implants broadens the potential clinical applications of PCD-CT, especially in scenarios involving complex endodontic retreatments or patients with extensive metal-based dental restorations and implants^23,35^.

Another intriguing possibility could be the opportunistic dental screening of otherwise indicated ultra-high resolution PCD-CT scans of the head and neck. Given that approximately 60% of the adult population in Europe has at least one root-filled tooth^36^, such opportunistic PCD-CT scans can provide important, high-quality endodontic cross-sectional images which can be used for potential further treatment decision. In detail, such scans could enable the systematic identification of subtle dental pathologies without the need for additional specialized imaging and repeated radiation exposure, thus improving overall patient care. The clinical relevance of the identified potential of PCD-CT for dental imaging is paramount. As PCD-CT becomes more increasingly established in many institutions, patients with dentomaxillofacial neoplasms or facial trauma will be scanned in PCD-CT more frequently. Thus, PCD-CT might allow optimized treatment planning based on already available emergency or staging examinations avoiding repeated X-ray images. In theory, the recent advances in detector technology might also enable the development of the next generation of CBCT with a photon-counting-detector as a dedicated modality for clinical implementation in dentistry.

While the results of this phantom study are promising, there are some limitations that need to be considered. Firstly, the use of an artificial bovine-teeth phantom setup, though anatomically representative, can neither replicate the complexity and heterogeneity of human dental anatomy, nor the clinical challenges such as patient movement or soft-tissue artifacts. Furthermore, the cylindrical, uniform artificial canals employed here lack the anatomical irregularities and calcifications frequently observed clinically. Based on the incidental finding of the drill tip, future studies should involve various filling materials, metal posts in clinical scenarios, and human teeth reflecting these complexities. Secondly, although CBCT showed substantially lower radiation dosages, the validity of this dosage-comparison between CBCT and PCD-CT in this study is very limited due to the non-anthropomorphic design. The effective dosage of PCD-CT examinations of dental structures needs to be investigated in further studies using clinical data as well as anthropomorphic phantoms. Noteworthy, our findings are device- and protocol-specific and should not be extrapolated to all CBCT systems as prior work shows notable inter-system variability for crack and fine-structure visibility, especially in the presence of high-density materials^37^. Additionally, as the CBCT image reconstructions in this study had voxel sizes of 150 μm, the Nyquist-limited practical resolution of dental CBCT potentially constrains the detectability of sub-voxel canals. Noteworthy, the precision analysis showed test retest variability below each modality’s in plane voxel size, indicating that width estimates at the smallest diameters are resolution limited rather than reader driven. Accordingly, a separate intraobserver reread was omitted. These precision metrics reflect combined repositioning and reader effects and do not isolate reader only variability, which is expected to be lower. Finally, as this is a feasibility-oriented phantom experiment, a formal clinical power calculation was not applicable. Although repeated, position-varied acquisitions provide multiple independent measurements and reduce scanner-position bias, anatomical variability and clinical factors remain unmodeled; thus, external validity is limited and should be addressed in larger, clinically powered studies.

In this phantom study, the null hypotheses for canal detectability and measurement accuracy were rejected: PCD-CT showed significantly higher detection rates of small accessory canals and lower measurement errors compared with dental CBCT. In contrast, the null hypothesis regarding blooming artifacts could not be rejected, as artifact widths varied between modalities without a consistent advantage for either scanner. In conclusion, PCD-CT might offer substantial improvements over dedicated dental CBCT, particularly in accurately detecting and measuring small anatomical structures in root-filled teeth without compromising image quality. These capabilities might allow for improved endodontic and periodontal imaging, thus enhancing patient outcomes through better-informed treatment planning and broadening diagnostic accuracy across dental imaging.

## Supplementary Information

Below is the link to the electronic supplementary material.

Supplementary Material 1

## Data Availability

The datasets generated during and/or analysed during the current study are available from the corresponding author on reasonable request.

## Abbreviations

CBCT: Cone beam CT
CT: Computed tomography
CTDI_vol_: Volume CT dose index
DLP: Dose-length product
DAP: Dose-area product
PCD: CT-photon-counting-detector CT
QIR: Quantum iterative reconstruction
RC: Root canal
ROI: Region-of-interest
