# Supplementary material for: Photon-counting CT outperforms dental CBCT in detecting small accessory canals in root-filled teeth in a phantom study
Source: Sci Rep. 2025 Oct 27;15:37352. doi: 10.1038/s41598-025-24439-0 (PMC12559294; doi:10.1038/s41598-025-24439-0)
Supplement: Supplementary file 1 — Supplementary Material 1 [file 41598_2025_24439_MOESM1_ESM.docx]

# Supplementary Figures:

**Supplemental Figure 1**

**
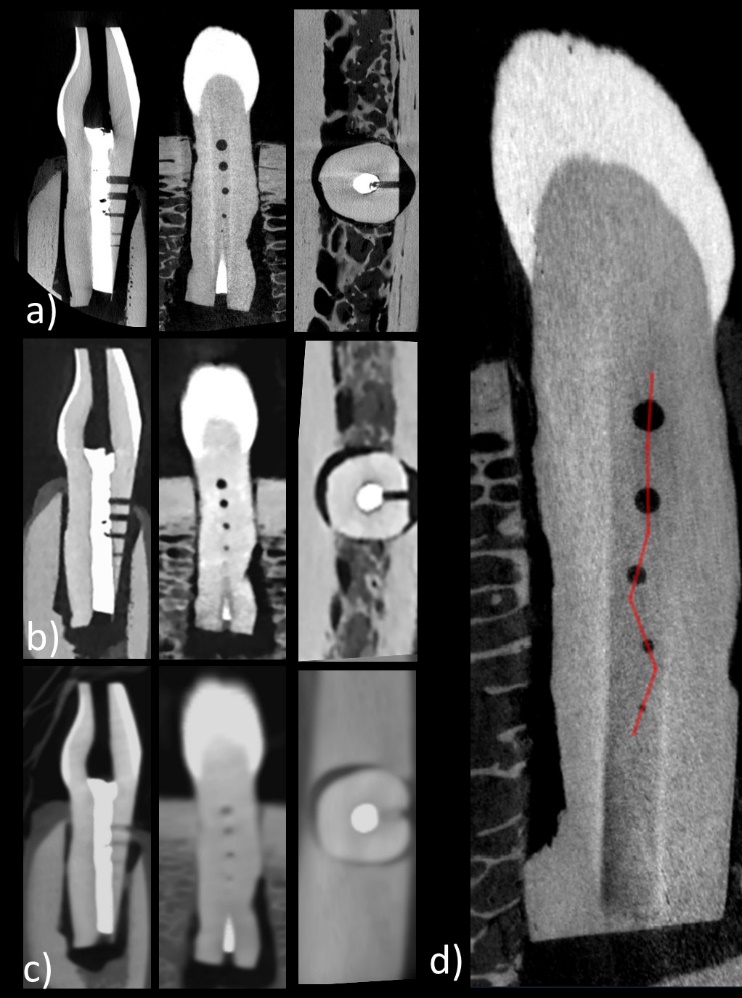
**

**Supplemental Figure 1**: Full-width-at-half-maximum measurement on multiplanar reconstructions of the root-filling in tooth 1.

The first row shows images acquired using photon-counting-detector CT (PCD-CT), the second row displays images from the cone-beam CT (CBCT). The red line was drawn through the filled main canal, centered at the mid-height between the first and second accessory canals, and in the exact direction of the first canal (with the largest diameter). The graphs on the left display the derived line-profiles of these examples.
